# Similar outcomes for antenatally or postnatally acquired haemorrhages

**DOI:** 10.1111/dmcn.14731

**Published:** 2020-11-15

**Authors:** Linda S de Vries, Johanna I P de Vries

**Affiliations:** ^1^ Department of Neonatology University Medical Center Utrecht Utrecht the Netherlands; ^2^ Department of Obstetrics and Gynaecology Amsterdam University Medical Center Amsterdam the Netherlands

## Abstract

This commentary is on the systematic review by Dunbar et al. on pages 144–155 of this issue.

The systematic review and individual patient data meta‐analysis of antenatally diagnosed fetal germinal matrix hemorrhage and intraventricular hemorrhage (GMH‐IVH) by Dunbar et al. is a welcome contribution to the literature.^1^ Until now, available data had mostly come from small case series.

In terms of both overall mortality and morbidity, the authors found outcomes not dissimilar to those reported for infants born very preterm diagnosed with perinatal and early postnatal onset of GMH‐IVH and its complications. This is particularly so for parenchymal haemorrhagic venous infarction (PVHI).

The meta‐analysis includes papers from 1982. With inevitable limitations on the quality of ultrasonography and no fetal magnetic resonance imaging (MRI) before the mid‐1990s, one does wonder whether data spanning almost 40 years are sufficiently uniform to be analysed together. Few papers will include data from fetal transfontanellar multiplanar (coronal and sagittal) sonographic brain imaging which was introduced around 1995 and recommended when brain anomalies are suspected in addition to standard transabdominal axial imaging. However, its application is still limited and therefore an updated guideline has been published recently.^2^ Unlike neonatal brain imaging, fetal brain MRI is not the criterion standard, but can be helpful when abnormalities are recognized with multiplanar sonography.

The primary outcome used was mortality, which will vary between countries for several reasons, not least differences in the upper age limit for termination of pregnancy. Fortunately, the authors also looked at neurodevelopmental outcome, though the median age was 12 months which is very early, especially for cognitive outcomes. Developmental delay was not described in any of the children with grades‐I/II GMH‐IVH. This may be due to mostly milder delays in infants with low‐grade haemorrhage not being detectable so early.

Another problem which may be dealt with in different ways is progressive ventricular dilatation after fetal haemorrhage. Almost all infants with a grade‐III IVH or PVHI were diagnosed as having hydrocephalus. Just over 40% of these infants were born preterm, but no mention is made whether this was spontaneous, due to induction of labour, or elective caesarean section. Some centres would consider early delivery by induction or elective caesarean section at 32 to 36 weeks’ gestation to allow earlier neurosurgical intervention, preventing prolonged increased pressure on the periventricular white matter. Importantly, recent studies have shown improved outcomes after early neurosurgical intervention.^3^ Other centres may allow the pregnancy to proceed until (near) term age and this could explain why the shunt rate among the infants with a severe fetal haemorrhage in this study was as high as 50%. Indeed, Dunbar et al. did note an association between in utero progression of hydrocephalus and the need for VP‐shunt placement. Results of intrauterine therapies are improving, with fetal endoscopic third ventriculostomy now even being used.^4^

Underlying genetic problems would not have been assessed in the early years of the study period, but now need to be considered as a cause for PVHI. *COL4A1* and *COL4A2* especially are increasingly recognized, even during pregnancy, and are known to carry a poorer prognosis than for an infant with a PVHI without an underlying genetic disorder. As all but one of the metabolic/genetic diagnoses made were in fetuses with PVHI, this group appears to be of special interest when considering additional investigations and a genetic workup is often recommended. Unfortunately, cerebellar haemorrhage, which may accompany larger supratentorial haemorrhage, was not included in this review, though a number of fetal case series exist.^5^

As antenatal GMH‐IVH and sequelae are not common problems, this paper may inspire international consortia to develop registers to help elucidate risk factors, collect outcome data, improve specific predictions of hemiplegia in association with PVHI for individual fetuses, and develop interventions.
